# Growth hormone prescribing and initial BMI SDS: Increased biochemical adverse effects and costs in obese children without additional gain in height

**DOI:** 10.1371/journal.pone.0181567

**Published:** 2017-07-17

**Authors:** Daniel B. Hawcutt, Jennifer Bellis, Victoria Price, Anne Povall, Paul Newland, Paul Richardson, Matthew Peak, Jo Blair

**Affiliations:** 1 Department of Women’s and Children’s Health, University of Liverpool, Liverpool, United Kingdom; 2 Paediatric Medicines Research Unit, Alder Hey Children’s Hospital, Liverpool, United Kingdom; 3 Research Department, Alder Hey Children’s NHS Foundation Trust Liverpool, United Kingdom; 4 Department of Biochemistry, Alder Hey Children’s NHS Foundation Trust, Liverpool, United Kingdom; 5 Department of Endocrinology, Alder Hey Children’s NHS Foundation Trust, Liverpool, United Kingdom; Chelsea and Westminster Hospital NHS Foundation Trust, UNITED KINGDOM

## Abstract

**Background:**

Recombinant human growth hormone (rhGH) treatment in children is usually prescribed using actual body weight. This may result in inappropriately high doses in obese children.

**Methods:**

Retrospective audit of all paediatric patients treated with rhGH 2010–14 at a tertiary paediatric hospital in the UK. Change in height SDS and IGF-I SDS during the first year of treatment was stratified by initial BMI SDS in a mixed cohort, and a subgroup of GH deficient (GHD) patients. Alternative doses for those BMI SDS ≥2.0 (Obese) were calculated using BSA, IBW and LBW.

**Results:**

354 patients (133 female) received rhGH, including 213 (60.2%) with GHD. Obesity was present in 40 patients (11.3%) of the unselected cohort, and 32 (15.0%) of the GHD cohort. For GHD patients, gain in height SDS was directly related to BMI SDS, except in obese patients (p<0.05). For both the entire cohort, and GHD patients only, IGF-1 SDS was significantly higher in obese patients (p<0.0001 for both groups). Cross sectional data identified 265 children receiving rhGH, 81 (30.5%) with a BMI-SDS ≥1.75. Alternate prescribing strategies for rhGH prescribing in obese patients suggest a saving of 27% - 38% annually.

**Conclusions:**

Gain in IGF-I SDS is greater in obese children, and is likely to be related to relatively higher doses of rhGH. Additional gain in height was not achieved at the higher doses administered to obese children. Alternative dosing strategies in the obese patient population should be examined in rigorous clinical trials.

## Introduction

In many paediatric populations worldwide the proportion of children who are overweight and obese is increasing, but there is wide geographical variation (from 16.9% in the USA to 0.5% in Denmark) [1, 2].

Obesity has direct effects on dosing any drug, increasing the proportion of the total body weight (TBW) composed of lipid, thereby providing a reservoir for lipophilic medications, while risking overdose of drugs contained within the intravascular compartment as this does not increase in proportion with the increase in weight [3]. Validated algorithms exist to manipulate a child’s actual weight to either an ideal or lean body weight, but these are not widely used within paediatrics [4]. Pharmacokinetic data in obese patients do not exist for the majority of drugs, although it is well understood that it will have substantial effects on the clearance and volume of distribution of many medicines [5]. In addition to the lack of pharmacokinetic data, there is little direct evidence as to how obesity affects the overall risk benefit of medications. rhGH offers a unique opportunity to examine this, as the population receiving it routinely have height and weight measured, and the positive outcome (height gain) and adverse effect (increase in IGF-1) are both routinely measured.

In non-obese adults, the volume of distribution of rhGH delivered exogenously has been calculated at approximately 78L [6], which is consistent with the majority of the drug being distributed in the total body water compartment (with the intravascular proportion predominantly bound to growth hormone binding protein [7]). Non-obese children have an increased total body water compared to adults, so the value will be slightly altered (likely decreased), while obese children have marked increases in free fat mass and mineral components rather than total body water **[8]**. Therefore, obese children receiving increased doses of rhGH derived from TBW are likely to have higher concentrations of rhGH in the total body water compartment than children of a similar height who are not obese.

Recently, concerns have been raised about the safety of rhGH therapy, specifically the long term oncological and cardiovascular effects. A meeting of the European Society of Paediatric Endocrinology, the Growth Hormone Research Society and the Pediatric Endocrine Society appraised these data recently [9]. It was recommended that insulin like growth factor 1 (IGF-1) levels should be maintained in the normal range [9], as there are no data reporting the safety of maintaining IGF-1 levels above the normal range. Conversely, for those in whom the growth response to treatment is poor, the position statement noted that the rhGH dose could be increased, recognising that IGF-1 levels may rise above the normal range, in order to achieve improved growth [9]. However, the rhGH doses required to achieve improved growth were very high (91 micrograms/kg daily) and the safety data relating to this practice are considered insufficient [10]. No recommendations for obesity were included.

The GH IGF-1 axis is profoundly affected by changes in BMI, with even modest changes in BMI affecting the activity of important binding proteins which result in an increase in GH sensitivity, and an increase in free, biologically active IGF-I [11–13], potentially making obese children particularly susceptible to adverse effects of high rhGH doses.

A randomised controlled trial of a prediction model (using a combination of biochemical and auxological data, including weight) against TBW based dosing did not identify any significant differences in the height achieved or dose administered [14]. However, the BMI SDS of the intervention group was -0.47 ± 1.07, and of the standard treatment arm -0.41 ± 0.97, meaning that the impact of obesity was not examined. A recent publication reported there was no significant difference in growth outcomes in girls with Turner syndrome treated with rhGH doses calculated according to TBW to those with doses calculated according to body surface area (BSA), although potential cost savings were identified [15].

The ideal dosing strategy in obese children has therefore yet to be determined. Measures of body size other than TBW may be more clinically appropriate [16], and could have significant financial implications.

In this study, the effects of rhGH doses calculated using TBW on gain in height-SDS and IGF-1 SDS during the first year of treatment were examined according to BMI SDS. This is the period over which greatest gain in height is observed, and is therefore the period at which differences between groups of patients are most likely to be identified. In addition, using cross sectional data, the doses used were compared to those that would have been prescribed if doses had been calculated using ideal body weight (IBW), lean body weight (LBW), and BSA. From these calculations we have estimated the approximate cost saving that would be achieved if these alternative measures of body size were used to calculate rhGH doses.

## Methods

### Setting

Data were collected from a specialist children’s hospital which provides tertiary paediatric endocrinology services with a catchment area covering approximately 2.7 million people (Merseyside, Cheshire and North Wales), including approximately 378,000 children and young people. Audit approved by Alder Hey Children's Hospital Audit Department. All data is routinely collected as part of clinical care.

### Patients

All patients who commenced treatment with rhGH between 2010 and 2014, were identified on our patient database. The following data were extracted from the appointment prior to starting rhGH treatment: clinical indication, gender, BMI-SDS, height-SDS, and IGF-1 SDS. IGF-1 SDS 1 year (+/-3 months) and height SDS 1 year (+/-2 months) following the start of treatment was also recorded. For patients in whom the height SDS was recorded less than ten months or more than 14 months after the start of treatment, height SDS at one year was derived from measurements before and after this time point.

Patients were studied in two cohorts: (1) an unselected cohort of patients with multiple diagnoses and (2) only those with GHD.

Height SDS and BMI SDS were derived from 2007 WHO growth data [17, 18]. Previously validated formulae [4] were used to calculate IBW and LBW for each child. Body surface area (m^2^) was calculated using the Boyd equation [19]. IGF-1 SDS were derived from data reported by Elminger et al [20].

### IGF-I assay

IGF-1 was measured using the Siemens Immulite 2000, using reagents supplied by Siemens Healthcare Diagnostic products Ltd, UK. The assay is a solid-phase, enzyme-labeled chemiluminescent immunometric assay. The performance of the assay was monitored using internal quality control and the inter and intra assay Coefficient of variation for the assay was less than 8%.

### Comparison of clinical outcomes for those with various BMI SDS at start of treatment

Patients were stratified according to BMI SDS: ≤-2.0, -1.99 to -1.01, -1.0 to 1.0, 1.01 to 1.99, and ≥2.0. Obesity was defined as BMI SDS ≥2.0.

In order to examine the relationship between TBW based prescribing and response to treatment, data from the first year of treatment were examined. In this unit, after the first year of treatment, rhGH doses are adjusted within the licensed dosing range, according to growth response and IGF-1 levels, with the intention of maintaining IGF-I levels within the normal range. To ascertain rhGH doses, after dose adjustment, and the cost implications of any changes in prescribing practice, a cross sectional approach was also employed using data from all patients receiving rhGH in October 2014.

For each patient with a BMI SDS BMI-SDS ≥2.0, the initial GH dose (mg/day) was divided by their TBW (kg) to obtain an ‘intended mg/kg dose’. This dose was then multiplied by a) IBW (kg) and b) LBW (kg) to calculate the mg/day dose based on these measures of body size. For all included patients, the ‘intended mg/kg dose’ was converted to an ‘intended mg/m^2^ dose’ using a conversion formula [21] which required the patient’s mg/kg/week dose, TBW (kg) and BSA (m^2^).

TBW, IBW and LBW mg/day doses were multiplied by 365 to give a dose (mg/year) for each patient. The mg/year dose was subsequently multiplied by a unit cost for the various brands of growth hormone to give the annual costs of treatment for each patient based on each of the three different parameters (BSA, TBW, IBW and LBW). These costs are the unit costs of 1mg of each of the brands of growth hormone available in the UK according to the British National Formulary for Children 2014/15 [22].

### Statistics

Statistics were undertaken using Microsoft Excel 2013.

## Results

### 1^st^ prescription of rhGH

Between 2010 and 2014 354 patients (133 females and 221 male) were prescribed rhGH for the first time. Patient demographics and indications for treatment are shown in Table 1, with additional anonymised data (S1 Table). Forty (11.3%) patients from the unselected cohort were obese (BMI-SDS ≥2.0) at the start of treatment, comprising 21 boys and 19 girls. From the GHD cohort, 32 (15.0%) were obese at the start of treatment.

**Table 1:** Patient demographics.

			Unselected cohort	Growth Hormone Deficiency cohort
	Number of patients		**354**	**213**
	Male (%)		**62.4**	**68.5**
	Age at start of rhGH treatment (mean years)		**12.27**	**12.25**
	Baseline BMI SDS (median)		**0.27**	**0.72**
		BMI SDS ≤-2.0 (n, %)	**22 (6.2%)**	**4 (1.9%)**
		BMI SDS -1.99 to -1.01 (n, %)	**52 (14.6%)**	**27 (12.7%)**
		BMI SDS 1.0 to -1.0 (n, %)	**174 (49.2%)**	**94 (44.1%)**
		BMI SDS 1.01 to 1.99 (n, %)	**66 (18.6%)**	**56 (26.3%)**
		BMI SDS ≥2.0 (n, %)	**40 (11.3%)**	**32 (15.0%)**
	Baseline Height SDS (median, range)		**-2.64 (-6.2 to 2.94)**	**-2.46 (-5.16 to 2.94)**
**Indication for Growth Hormone (rhGH)**				
	Growth Hormone Deficiency		**213**	
	IUGR/SGA		**54**	
	Turners Syndrome		**25**	
	Idiopathic Short Stature		**16**	
	Russell-Silver Syndrome		**15**	
	Chronic Kidney Disease		**10**	
	Prader Willi Syndrome		**9**	
	Noonan’s Syndrome		**5**	
	Other		**7**	

Height SDS was documented for all patients one year after the start of rhGH treatment. IGF-1 SDS was documented in 279 patients (78.8%), including 182 (85.4%) of those with a diagnosis of GHD. The patients with missing data for IGF-1 SDS one year following the start of treatment were distributed evenly throughout the BMI-SDS categories. Across the entire cohort, evaluable data for change in IGF-1 SDS were available as follows: ≤-2.0 n = 17 (77.2%); -1.01 to -1.99 n = 42 (80.8%); 1.0 to -1.0 n = 138 (79.3%); 1.99 to 1.01 n = 50 (75.8%); ≥2.0 n = 32 (80.09%). Mean height SDS and IGF-1 SDS at the start of treatment are shown in Table 2.

**Table 2:** Comparison of the baseline height SDS and IGF-1 SDS scores for the children in each BMI-SDS cohort for both the entire group and GHD subgroup.

	Entire Cohort		Growth Hormone Deficiency	
BMI SDS range	Mean Baseline Height SDS (n = 354)	Mean Baseline IGF-1 SDS (n = 289)	Mean Baseline Height SDS (n = 213)	Mean Baseline IGF-1 SDS (n = 186)
≤2.0	-3.15	-2.17	-2.78	-2.88
-1.01 to -1.99	-2.96	-2.02	-2.71	-2.24
1.0 to -1.0	-2.63	-1.80	-2.404	-2.02
1.01 to 1.99	-2.323	-1.88	-2.31	-1.99
≥2.0	-1.35	-1.87	-1.31	-2.18
P value (ANOVA)	**p<0.0001**	p = 0.84	**p<0.0001**	p = 0.90

The children within the lowest BMI-SDS category (≤-2.0) were shorter at the initiation of treatment than those children with higher BMI-SDS scores, in both the unselected cohort and GHD sub-group (p<0.0001 for both) (Table 2). The children in the highest BMI-SDS category have an excess of brain tumour diagnoses (13/40 unselected, 13/32 GHD), reflecting the known association between obesity and structural defects of the hypothalamic pituitary axis. The growth of patients with known midline abnormalities of the brain is monitored closely, and growth hormone testing is undertaken when the height velocity falls below 0SD. The mean height SDS at the start of treatment for children treated following brain tumours was -0.77, greater than for other groups (Table 2).

Baseline IGF-1 SDS did not differ between any of the BMI-SDS categories in either the unselected cohort (p = 0.84) or GHD subgroup (p = 0.90) (Table 2). After 1 year, IGF-1 SDS was ≥2.0 in 31/354 patients (8.6%) in the unselected cohort, including 11/32 obese patients (34.4%) with evaluable 1 year IGF-1 SDS measurements. Within the GHD subgroup, IGF-1 SDS was ≥2.0 in 19/213 patients (8.9%), including 8/27 obese patients (29.6%) with evaluable 1 year IGF01 SDS measurements.

**Fig 1:**
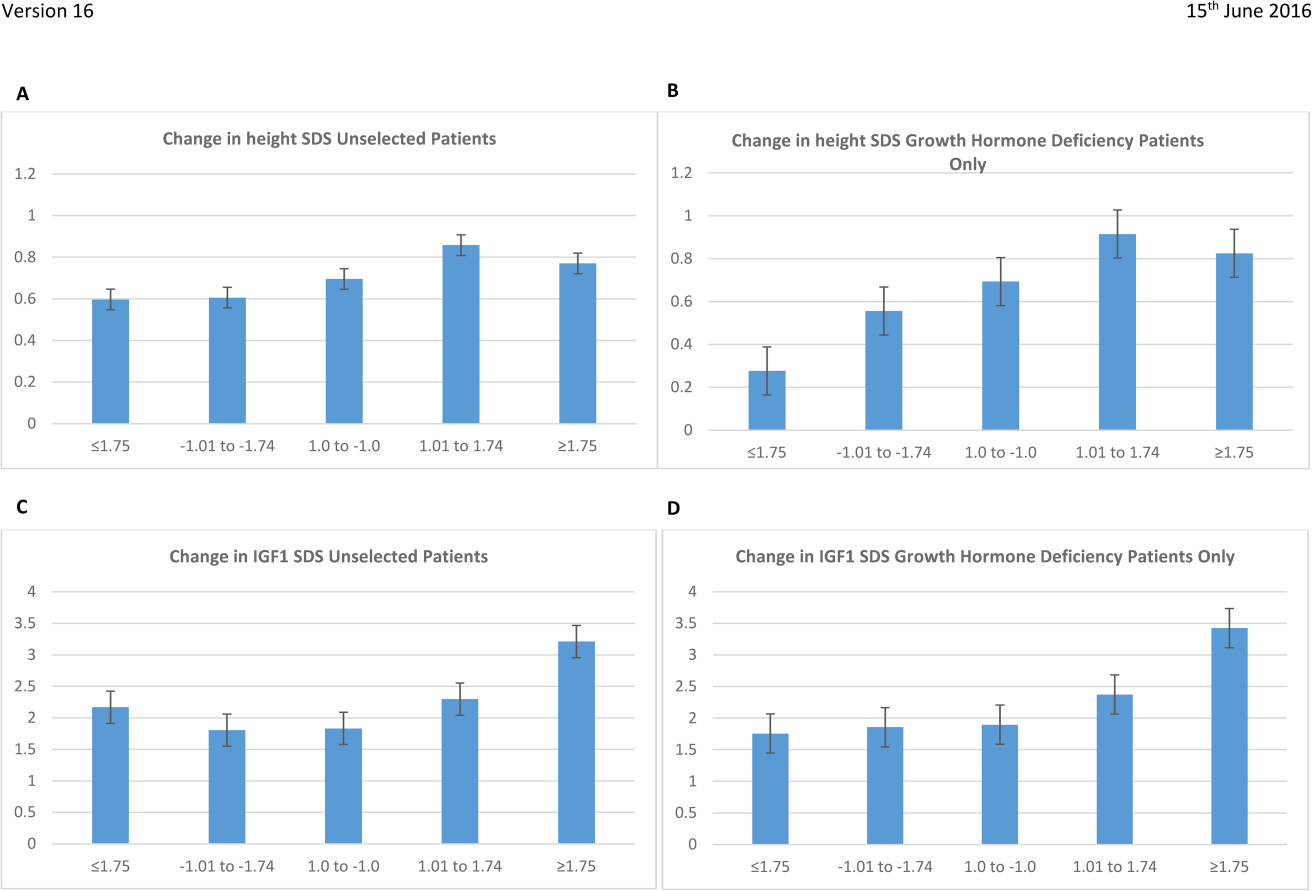
A: Change in height-SDS for an unselected group of patients receiving rhGH after one year of treatment against BMI-SDS at initiation of prescription. Change in growth p = 0.041 (ANOVA) B: Change in height-SDS for patients with growth hormone deficiency and receiving rhGH after one year of treatment against BMI-SDS at initiation of prescription. Change in growth p = 0.005 (ANOVA) C: Change in IGF-1-SDS after one year of treatment against BMI-SDS at initial rhGH prescription for an unselected group of patients. Change in IGF-1 p<0.0001 (ANOVA) D: Change in IGF-1-SDS after one year of treatment against BMI-SDS at initial rhGH prescription for patients with growth hormone deficiency. Change in IGF-1 p<0.0001 (ANOVA).

Changes in height-SDS and IGF-1-SDS after one year for both cohorts for each BMI-SDS category are shown in Fig 1. Across the unselected cohort, changes in the height-SDS and IGF-1-SDS with increasing BMI-SDS were statistically significant (p = 0.041 and p<0.0001 respectively, ANOVA). For those with a diagnosis of GHD, changes in height-SDS and IGF-1-SDS with increasing BMI-SDS were also statistically significant (p = 0.005 and p<0.0001 respectively, ANOVA). For the unselected cohort, the mean change IGF-1-SDS was 81.3% greater in those within the cohort of BMI ≥2.0 compared to the cohort 1.0 to -1.0. For those with GHD, the mean change in IGF-1-SDS was 90.5% greater in those within the cohort of BMI ≥2.0 compared to the cohort 1.0 to -1.0.

### Prescribing of rhGH in October 2014

In October 2014, we identified 265 children receiving treatment with rhGH. Of these, 67 (25.3%) were obese (male 39, female 28). The doses received by these children using TBW, BSA, IBW, and LBW prescribing are shown in Table 3.

**Table 3:** Characteristics of patients BMI-SDS ≥2.0 being treated with growth hormone in October 2014.

Cohort		Median age, Q1, Q3 (years)	Median BMI SDS, Q1, Q3	Median growth hormone dose, Q1, Q3 (mg/day)			
				Calculated using TBW	Equivalent BSA dose, p value	Calculated using IBW, p value	Calculated using LBW, p value
**BMI SDS ≥ 2.0**	**Males** **(n = 39)**	12.33 (8.59, 15.88)	2.71 (2.43, 3.25)	1.10 (0.60, 1.75)	1.07 (0.58, 1.68), p = 0.99	0.72 (0.43, 1.05), **p = 0.001**	0.80 (0.48, 1.25), **p = 0.023**
	**Females** **(n = 28)**	11.16 (8.76, 14.48)	2.62 (2.33, 3.21)	1.20 (0.80, 1.80)	1.19 (0.77, 1.71), p = 0.92	0.76 (0.53, 1.09), **p = 0.0009**	0.88 (0.63, 1.28), **p = 0.02**

Comparison of prescribed growth hormone doses (based on mg/kg initial dosing, but potentially modified to response/IGF-1 results) in a cross section of patients in October 2014, against with equivalent body surface area doses. P values calculated using Mann-Whitney U test), significant (<p<0.05) shown in bold.

For patients whose BMI-SDS ≥2.0, as expected the median daily dose of rhGH is reduced when the dose is calculated using IBW or LBW instead of TBW for both males and females. The dose reduction is largest when the dose is calculated using IBW.

At our centre, considerable annual cost savings would be realised if rhGH doses were calculated using either IBW or LBW instead of TBW for obese patients. Percentage cost savings for patients with TBW>IBW and obese patients are shown, as well as actual cost saving per brand (assuming 100% of patients are using that brand) are shown in Table 4.

**Table 4:** Comparison of costs for growth hormone doses calculated using total body weight (TBW), ideal body weight (IBW) and lean body weight (LBW).

	Brand of Growth Hormone	Change in growth hormone prescribed annually using IBW (%)	Predicted costs savings prescribing using IBW	Change in growth hormone prescribed annually using LBW (%)	Predicted cost savings prescribing using LBW
**Individuals BMI SDS ≥ 2.0** **(n = 67)**	**Genotropin**^**®**^ **(Pfizer Ltd)**	**-37.7%**	£195,285 / $254,405 / €227,754	**-26.8%**	£138,652 / $180,627 / €161,668
	**Humatrope**^**®**^ **(Eli Lilly and Company Ltd)**		£202,135 / $263,415 / €235,614		£143,516 / $187,025 / €167,291
	**Norditropin® Nordiflex** **(Novo Nordisk Ltd)**		£260,306 / $339,151 / €303,936		£184,817 / $240,712/ €215,794
	**Norditropin® SimpleXx®** **(Novo Nordisk Ltd)**		£238,857 / $311,096 / €278,829		£169,588 / $220,929 / €197,968
	**NutropinAq**^**®**^ **(Ipsen Ltd)**		£226,640 / $295,297 / €264,377		£160,915 / $209,661 / €187,708
	**Omnitrope®** **(Sandoz Ltd)**		£165,639 / $215,852 / €193,258		£117,603 / $153,254 / €137,212
	**Saizen**^**®**^ **(Merck Serono Ltd)**		£260,306 / $339,218 / €303,936		£184,817 / $240,844 / €215,794
	**Zomacton**^**®**^ **(Ferring Pharmaceuticals Ltd)**		£223,472 / $291,244 / €260,567		£158,665 / $206,783 / €184,949

## Discussion

We have demonstrated that in gain in height-SDS and IGF-1 SDS during the first year of rhGH treatment is related to BMI-SDS at the start of treatment in both an unselected cohort of children treated with rhGH and those with GHD. In both groups, the change in height-SDS appears to show a ‘ceiling effect’ whereby no additional height is attained despite a TBW-derived dose being higher. However, there was also a suggestion of reduced height gain in children with GHD who have the lowest BMI-SDS. A different pattern has been shown with changes in IGF-1 SDS, where doses for those with BMI-SDS >1.0, and particularly ≥ 2.0, are associated with greater increases in the IGF-1 SDS one year after the start of rhGH treatment for both the unselected cohort and those with GHD. Compared to children with a BMI-SDS between -1.0 to +1.0, those with a BMI-SDS ≥2.0 had at least an 80% greater increase in IGF-1 SDS. This is the first data we are aware of to quantify the differential effect of obesity on efficacy and adverse effects in a population, and suggests that alternative dosing strategies need to be explored for rhGH.

The cross sectional data have shown that a considerable proportion of children treated with rhGH in this centre are obese. This is to be expected where levels of obesity are high in the background population, and where some diagnoses are associated with an increased risk of overweight and obesity, in particular those with Prader Willi Syndrome, girls with Turner Syndrome and those lesions affecting the midline structures of the brain.

A previous study did compare the cost effectiveness of rhGH prescribing. It compared BSA–based dosing with a TBW-based dosing regimen in girls with Turner’s syndrome, and found the BSA-based regimen was more cost-effective and potentially as effective as the TBW-based regimen [15]. Interestingly, rhGH doses based on BSA were not significantly different to rhGH doses based on TBW in those with a BMI-SDS ≥2.0 in this cohort.

There has also been a previous publication [21], relating growth response to rhGH treatment to BMI, an inverse relationship was reported between BMI and first year growth response, in contrast to our data in which the converse is true. The authors of this paper commented that this was likely to reflect the older age of patients in the heaviest cohort, as growth response is related to age at start of treatment. Furthermore, as children became heavier, it was the practice of clinicians prescribing GH to this population, to use doses that were derived from BSA rather than by mg/kg/day, resulting in a lower rhGH dose per kg than in the lighter patients. In our population of patients, a consistent approach to prescribing has been used across the range of BMI, allowing us to observe the effect of BMI more clearly. Our data relating to growth response are further strengthened by the relationship between IGF-I SDS and BMI.

Treatment with rhGH has a long and reassuring safety record. An increase in mortality in adults treated with GH during childhood has been reported by some investigators [23], but not others [24]. Nevertheless, an association between IGF-1 levels in the upper range in normal populations is associated with an increase in some common cancers, and it therefore makes sense to minimise the risk of GH treated patients being exposed to unduly high IGF-1 levels [25], a position re-iterated by the recent international growth hormone safety workshop [9]. It may be that any additional risks that obese children may be exposed to, due to the relatively higher doses of rhGH they receive, are masked by the excellent safety record in non-obese children.

It has been noted that changes in the GH-IGF-1 axis occur in obese children, which increases their sensitivity to GH and levels of free IGF-I (9–11). It is important to note that standard laboratory methods measure total, rather than free IGF-1. Given the changes that occur in the GH-IGF-1 axis in obese children, the true magnitude of the effect of TBW based dosing on IGF-I in obese children will only be revealed by measuring free IGF-1.

A number of factors are known to influence growth response to growth hormone treatment, including age at start of treatment, genetic height potential, height at start of treatment and growth hormone peak on a growth hormone stimulation test [26]. It is likely that genetic determinants of growth hormone sensitivity also influence growth response. In our unselected cohort of patients, there are numerous diagnoses included, and these may affect the response to treatment with rhGH. Formal dose response studies have clearly described a relationship between gain in height and growth hormone dose [27, 28], and high and low dose IGF-I generation tests also relate GH dose to increment in IGF-I [29]. Greater weight and higher growth hormone dose have been identified as predictors of first year growth response in previous cohorts of patients [26]. We speculate that it is the relatively higher doses of GH used to treat obese patients in this cohort that accounts for the greater gain in height and IGF-I SDS.

Altered IGF-I bioactivity has been reported in adolescent patients with Prader-Willi syndrome, a condition often associated with obesity, during treatment with growth hormone [30]. In these patients, IGF-I concentrations frequently increase above the normal range during growth hormone treatment leading to concerns regarding treatment safety. However, a recent study demonstrated that the bioactivity of IGF-I is reduced, as a greater proportion of IGF-I circulates bound to the ternary complex when compared to healthy control subjects. To date it is not known whether this alteration in IGF-I binding is a feature of obesity or Prader Willi syndrome, but it is noteworthy that the cohort of patients reported in this study were not obese (mean BMI SDS 1.2, inter quartile range 0.2–1.7). Furthermore, in multiple regression analysis, IGF-I bioactivity was not related to BMI SDS at the time of time of sampling. Nevertheless, this is an interesting phenomenon that should be examined in obese patients treated with GH for other indications.

Our data raise the specific question as to whether the current strategy of prescribing rhGH according to TBW is appropriate and could equivalent growth (and improved safety) be achieved with lower doses calculated using LBW or IBW? It is our view that the target of rhGH treatment should be to achieve a final adult height (FAH) that is as close as possible to the target height (calculated from parental heights), rather than simply achieving the greatest possible FAH. Therefore, while the lower doses of rhGH that would be prescribed from doses calculated from measures of body size other than TBW may be associated with modest reductions in FAH, this may be acceptable if the FAH remains in the target range. This question should now be addressed in a carefully designed clinical trial.

In addition, it highlights how the presence of obesity can alter the benefit:risk profile of a medication if dosing is undertaken using the standard mg/kg system, and there may be better alternatives that could be used instead [16]. rhGH is unusual in that there are few other medicines where both the intended therapeutic gain and main adverse effect can be measured as specifically. However, it represents an opportunity to improve dosing

A limitation of this work is that it is a single centre study, with limited numbers, and our data may not be generalizable. Cost calculations are based on UK formulary prices, and will vary internationally, but are sufficiently indicative for our cost modelling assumptions.

Further research should focus on confirming these findings at other centres to demonstrate their generalizability and the potential for NHS-wide cost savings. Subsequently a randomised controlled study comparing patients treated with GH in doses calculated by different measurements of body mass should be undertaken. This study should compare the clinical outcomes for patients on the various regimens to determine optimal safety, clinical and cost effectiveness.

## Conclusion

Gain in height and IGF-I SDS is related to BMI-SDS at the initiation of rhGH therapy, and may be related to the relatively higher doses of rhGH prescribed to obese children. Additional gain in height was not achieved at the higher doses administered to obese children. If dosing according to IBW or LBW gives similar growth responses across the range of BMI, it may be possible to reduce the potential risks of high IGF-I levels in obese children, while achieving a considerable cost saving.

## Supporting information

S1 Table Anonymised individual patient data. (PDF)
