# Supplementary material for: Growth hormone prescribing and initial BMI SDS: Increased biochemical adverse effects and costs in obese children without additional gain in height
Source: PLoS One. 2017 Jul 17;12(7):e0181567. doi: 10.1371/journal.pone.0181567 (PMC5513545; doi:10.1371/journal.pone.0181567)
Supplement: S1 Table — (PDF) [file pone.0181567.s001.pdf]

Table S1

Audit number	Baseline BMI SDS	Baseline Ht SDS	Baseline IGF1 SDS	1 yr Ht SDS	1 yr IGF1 SDS	Change in Height SDS	Change in IGF1 SDS
1	-4.38	-1.31	-2.85	-1.16	1.3	0.15	4.15
2	-4.35	-2.59	-2.71	-1.8	0.84	0.79	3.55
3	-3.35	-3.97	-0.84	-3.52	0.03	0.45	0.87
4	-3.17	-3.07	-0.88	-1.32	0.61	1.75	1.49
5	-3.01	-3.49	-2.27	-2.22	0.84	1.27	3.11
6	-2.99	-3.11	-2.28	-2.78	-1.75	0.33	0.53
7	-2.87	-2.2	-2.54	-1.74	-0.18	0.46	2.36
8	-2.86	-3.04	-0.33	-2.6	0.55	0.44	0.88
9	-2.85	-4.64	-0.79	-3.33	1.93	1.31	2.72
10	-2.8	-4.09	-1.06	-3.4	-1.27	0.69	-0.21
11	-2.78	-4.08	-3.14	-3.05	-1.35	1.03	1.79
12	-2.71	-2.5	-1.15	-2.52	-0.51	-0.02	0.64
13	-2.65	-3.33	-4.69	-2.85	-2.97	0.48	1.72
14	-2.5	-3.55		-2.8		0.75	
15	-2.47	-2.72	-2.82	-1.88	2.49	0.84	5.31
16	-2.44	-1.87		-1.19	-0.28	0.68	
17	-2.33	-3.35		-3	-0.09	0.35	
18	-2.27	-3.27	-1.17	-2.95	0.29	0.32	1.46
19	-2.2	-2.97	-4.33	-2.66	2.12	0.31	6.45
20	-2.16	-2.68	-2.96	-2.56	-1.2	0.12	1.76
21	-2.13	-3.39		-2.32		1.07	
22	-2.09	-4.16		-3	-0.23	1.16	
23	-1.96	-3.25	-1.49	-2.56	-0.04	0.69	1.45
24	-1.91	-4.2	-2.21	-4.06	-1.61	0.14	0.6
25	-1.88	-2.58	-3.99	-2.48	0.23	0.1	4.22
26	-1.86	-2.61	-0.61	-2.03	-0.69	0.58	-0.08
27	-1.83	-3.05	-4.72	-3.34	-1.77	-0.29	2.95
28	-1.78	-2.91		-2.15	0.6	0.76	
29	-1.73	-3.37	-2.73	-2.12	0.17	1.25	2.9
30	-1.72	-1.72	-3.08	-1.58	-1.13	0.14	1.95
31	-1.71	-3.66		-2.94	3.19	0.72	
32	-1.7	-1.57	-0.2	-0.4	1.56	1.17	1.76
33	-1.7	-2.06	0.08	-1.79	1.07	0.27	0.99
34	-1.68	-2.94	-2.97	-2.38	0.06	0.56	3.03
35	-1.68	-1.54	-1.53	-0.77	0.27	0.77	1.8
36	-1.66	-2.31		-1.56	2.33	0.75	

37	-1.63	-2.46	-0.78	-2.13	-0.22	0.33	0.56
38	-1.62	-2.96	-3.91	-2.74	-2.67	0.22	1.24
39	-1.61	-2.46	-4.08	-1.75	-0.67	0.71	3.41
40	-1.58	-2.18	-2.04	-2.21	-0.22	-0.03	1.82
41	-1.54	-2.97	-1.43	-2.19	1.6	0.78	3.03
42	-1.48	-4.67		-4.16	0.04	0.51	
43	-1.44	-6.2		-4.08	0.78	2.12	
44	-1.42	-2.86	-0.91	-2.18	0.28	0.68	1.19
45	-1.38	-3.34	-0.46	-3.15	0.49	0.19	0.95
46	-1.3	-2.24	-1.5	-2.05	0.02	0.19	1.52
47	-1.3	-1.86	0.05	-1.87	-1.45	-0.01	-1.5
48	-1.28	-3.14	-2.23	-2.41	0.6	0.73	2.83
49	-1.27	-3.86	-5.84	-3.06	-2.54	0.8	3.3
50	-1.27	-3.1	-2.07	-2.4	-0.45	0.7	1.62
51	-1.27	-3.17	-0.66	-2.52	0.66	0.65	1.32
52	-1.25	-3.45	-2.84	-2.75		0.7	
53	-1.22	-3.16	-1.02	-2.38	0.53	0.78	1.55
54	-1.22	-2.66	-1.59	-2.28	-0.38	0.38	1.21
55	-1.21	-2.8	-2.11	-2.18	0.02	0.62	2.13
56	-1.2	-2.77	-2.08	-2.1	1.4	0.67	3.48
57	-1.2	-2.3	-2.11	-1.58	-0.75	0.72	1.36
58	-1.19	-3.98	-1.75	-3.4	1.08	0.58	2.83
59	-1.17	-3.56	-1.81	-2.71	3.23	0.85	5.04
60	-1.16	-2.56	-1.89	-2.18	0.36	0.38	2.25
61	-1.15	-3.18	-1.97	-2.82	-0.31	0.36	1.66
62	-1.15	-2.97	-3.39	-2.21	-2.85	0.76	0.54
63	-1.15	-3.19		-2.33	0.54	0.86	
64	-1.15	-1.64		-1.23	0.16	0.41	
65	-1.14	-2.71	-4.18	-2.47	-2.31	0.24	1.87
66	-1.08	-2.15	-4.01	-1.7	-1.19	0.45	2.82
67	-1.08	-2.65	-0.61	-2.36	0.45	0.29	1.06
68	-1.08	-3.85		-3.38		0.47	
69	-1.07	-3.15	1.82	-2.8	0.36	0.35	-1.46
70	-1.05	-3.48	-2.67	-2.21	0.87	1.27	3.54
71	-1.05	-4.03	-0.77	-3.39	0.27	0.64	1.04
72	-1.03	-2.04		-1.77	-0.69	0.27	
73	-1.01	-4.03	-3.05	-2.92	-1.61	1.11	1.44
74	-1.01	-2.41	-1.51	-1.92	-0.77	0.49	0.74
75	-0.99	-1.65	-2.86	-1.23	0.11	0.42	2.97

76	-0.99	-1.72	-0.39	-0.97	1.07	0.75	1.46
77	-0.95	-2.85	-3.02	-2.71	-2.64	0.14	0.38
78	-0.94	-1.58	-0.97	-1.63	-0.19	-0.05	0.78
79	-0.94	-3.54	-3.14	-2.51	-2.55	1.03	0.59
80	-0.9	-3.62	-0.26	-2.45	1.01	1.17	1.27
81	-0.89	-2.98	-1.96	-2.72	-0.36	0.26	1.6
82	-0.89	-4.25	-2.13	-2.6	-1.08	1.65	1.05
83	-0.88	-4.13	-3.02	-3.75	-0.48	0.38	2.54
84	-0.87	-2.19	-4.64	-1.27	0.08	0.92	4.72
85	-0.87	-1.91		-0.91		1	
86	-0.85	-3.18	-2.72	-2.81	-0.36	0.37	2.36
87	-0.85	-2.07	-1.73	-0.64	0.01	1.43	1.74
88	-0.82	-2.52	-0.31	-1.8		0.72	
89	-0.76	-2.58	-3.55	-2.04	1	0.54	4.55
90	-0.76	-3.87	-0.56	-2.73	0.92	1.14	1.48
91	-0.75	-1.84		-1.14	-1.1	0.7	
92	-0.75	-3.01		-2.57		0.44	
93	-0.73	-2.76	-2.1	-2.31	0.16	0.45	2.26
94	-0.71	-2.26	-2.24	-1.77	-0.03	0.49	2.21
95	-0.69	-3.05	-0.36	-0.96	0.28	2.09	0.64
96	-0.68	-0.88	-1.7	-0.99	0	-0.11	1.7
97	-0.66	-4.06		-3.34	-0.95	0.72	
98	-0.64	-1.18	-3.69	-0.66	-0.12	0.52	3.57
99	-0.64	-3.51	-2.28	-2.95	0.24	0.56	2.52
100	-0.64	-2.3	-1.85	-1.84	0.42	0.46	2.27
101	-0.62	-2.45	-0.86	-1.29	2.82	1.16	3.68
102	-0.61	-2.14	-2.33	-0.94	-0.71	1.2	1.62
103	-0.61	-2.36	-1.08	-1.81	0.27	0.55	1.35
104	-0.61	-2	-0.41	-1.73	-0.57	0.27	-0.16
105	-0.6	-2.99	1.24	-3.02	2.13	-0.03	0.89
106	-0.6	-3.33	0.31	-2.71	-1.26	0.62	-1.57
107	-0.59	-2.6		-2	-0.17	0.6	
108	-0.58	-2.1	-1.13	-2	2.24	0.1	3.37
109	-0.56	-1.9	-2.49	-1.29	0.92	0.61	3.41
110	-0.56	-0.97	-1.12	-0.52	1.32	0.45	2.44
111	-0.55	-2.83	-2.24	-1.64		1.19	
112	-0.54	-2.78	-2.8	-2.24	-1.56	0.54	1.24
113	-0.53	-3.08	-5.02	-2.6	-3.69	0.48	1.33
114	-0.53	-3.54	-2.77	-3.12	-1.74	0.42	1.03

115	-0.52	-1.86	-2.26	-0.46	0.02	1.4	2.28
116	-0.52	-2.24	-1.78	-2.49	0.11	-0.25	1.89
117	-0.52	-2.64	-2.51	-2.12	-2.41	0.52	0.1
118	-0.48	-2.31	-2.06	-1.13	-1.14	1.18	0.92
119	-0.47	-2.69	-2.21	-2.1	-1.69	0.59	0.52
120	-0.47	-3.08	-0.57	-2.6	-0.78	0.48	-0.21
121	-0.45	-2.55	-0.12	-2.25	0.98	0.3	1.1
122	-0.42	-3.87	-0.89	-3.09	-0.35	0.78	0.54
123	-0.39	-3.89	-1.16	-3.05	-0.44	0.84	0.72
124	-0.37	-3.9	-2.99	-3.39	0.49	0.51	3.48
125	-0.37	-3.03	-1.9	-2.53	0.66	0.5	2.56
126	-0.37	-2.74		-2.19		0.55	
127	-0.35	-1.15	-4.68	-0.04	-0.97	1.11	3.71
128	-0.34	-2.28	-3.06	-1.56	-0.59	0.72	2.47
129	-0.34	-2.63	-1.04	-2.3	1.13	0.33	2.17
130	-0.34	-3.74		-4.04		-0.3	
131	-0.32	-2.56	-0.85	-2.02	0.26	0.54	1.11
132	-0.32	-3.87	0.2	-2.96	1.22	0.91	1.02
133	-0.29	-3.03	-3.37	-2.57	1.16	0.46	4.53
134	-0.29	-2.57	-0.91	-1.76	0.85	0.81	1.76
135	-0.29	-2.99		-1.37		1.62	
136	-0.28	-2.91	-3.9	-2.81	0.21	0.1	4.11
137	-0.28	-0.94	-1.44	-0.89	0.25	0.05	1.69
138	-0.27	-2.32	-3.49	-1.5	-1.06	0.82	2.43
139	-0.27	-3.31	-3.21	-2.65	-1.79	0.66	1.42
140	-0.27	-1.96	-1.22	-1.87	-0.05	0.09	1.17
141	-0.27	-1.39	-1.48	-1.28	-1.89	0.11	-0.41
142	-0.26	-4.92	-1.17	-3.51	-0.58	1.41	0.59
143	-0.23	-2.61	-1.67	-2.14	0.66	0.47	2.33
144	-0.21	-3.19	-0.99	-2.07	-0.97	1.12	0.02
145	-0.2	-1.55		-0.59	0.85	0.96	
146	-0.19	-2.62	-3.17	-1.34	0.25	1.28	3.42
147	-0.19	-3.14	-1.55	-2.39	-0.07	0.75	1.48
148	-0.18	-2.41	-0.6	-1.93		0.48	
149	-0.17	-3.27		-1.79		1.48	
150	-0.16	-2.01	0.38	-1.43	-0.43	0.58	-0.81
151	-0.14	-3.99	-3.13	-3	-3.36	0.99	-0.23
152	-0.12	-2.15	-2.36	-1.51	1.03	0.64	3.39
153	-0.11	-4.71		-3.63		1.08	

154	-0.07	-2.61	-0.81	-2.26	-0.17	0.35	0.64
155	-0.05	-3.2	-2.84	-2.42	-0.97	0.78	1.87
156	-0.05	-3.4		-1.86	1.37	1.54	
157	-0.04	-2.02		-1.31	0.38	0.71	
158	-0.02	-3.88	-3.36	-2.22	-1.49	1.66	1.87
159	-0.02	-2.89	-0.55	-2.57	1.09	0.32	1.64
160	-0.01	-3.65	-0.8	-3.05	1.41	0.6	2.21
161	-0.01	-3.4	0.43	-2.61	1.64	0.79	1.21
162	-0.01	-3.65	-1.31	-3.05	-0.13	0.6	1.18
163	-0.01	-3.45	0.76	-2.94		0.51	
164	0.01	-3.52	-3.64	-2.64	-0.74	0.88	2.9
165	0.01	-3.35	-2.5	-2.45	-0.08	0.9	2.42
166	0.02	-2.1	-3.15	-0.3	1.07	1.8	4.22
167	0.02	-3.25		-2.94	-0.86	0.31	
168	0.03	-1.73	-0.99	-1.42	-0.67	0.31	0.32
169	0.09	-2.34	-0.06	-1.85	1.65	0.49	1.71
170	0.11	-3.59	-2.22	-2.82	1.23	0.77	3.45
171	0.11	-2.84	-2.37	-2.03	0.59	0.81	2.96
172	0.16	-2.7	-0.14	-1.5	0.24	1.2	0.38
173	0.18	-2.7	-0.69	-1.96	2.21	0.74	2.9
174	0.21	-2.43	-3.67	-1.87	-1.85	0.56	1.82
175	0.22	-4.57	-3.04	-3.69	-2.58	0.88	0.46
176	0.23	-2.23	-1.97	-2.06	0.55	0.17	2.52
177	0.25	-2.83		-2.24	-0.31	0.59	
178	0.29	-3.57	-2.85	-2.57	0.51	1	3.36
179	0.29	-1.56	-1.72	-1.1	1.4	0.46	3.12
180	0.29	-1.55	0.02	-0.66	0.09	0.89	0.07
181	0.3	-2.78	-0.38	-2.54	0.72	0.24	1.1
182	0.31	-1.72	-2.81	-0.99	0.16	0.73	2.97
183	0.31	-2.72	-2.33	-1.76	-0.15	0.96	2.18
184	0.32	-3.55	-3.85	-3.25	-1.06	0.3	2.79
185	0.32	-2.63	-1.73	-2.24	0.29	0.39	2.02
186	0.32	-4.31		-2.52		1.79	
187	0.33	-3.06	-1.74	-2.02	0.45	1.04	2.19
188	0.33	-3.49	-3.4	-2.83	-3.61	0.66	-0.21
189	0.33	-2.96		-2.18	0.46	0.78	
190	0.33	-2.65		-2.23		0.42	
191	0.35	-1.47	-1.33	-1.1	-0.29	0.37	1.04
192	0.35	-3.05		-2.41	-2.99	0.64	

193	0.36	-1.86		-0.92		0.94	
194	0.37	-2.13	-1.01	-1.54	1.22	0.59	2.23
195	0.38	-3.48	-3.14	-2.23	-0.16	1.25	2.98
196	0.43	-0.73	-2.79	-0.74	-0.02	-0.01	2.77
197	0.43	-1.49	-1.62	-1.38	-0.08	0.11	1.54
198	0.43	-3.27		-2.51	0.24	0.76	
199	0.44	-3.09	-3.34	-2.09	-0.78	1	2.56
200	0.45	-3.76	-2.95	-2.73	-0.96	1.03	1.99
201	0.48	-3.11	-3.81	-2.57	0.98	0.54	4.79
202	0.48	-2.03	-0.57	-1.54	0.54	0.49	1.11
203	0.48	-3.2		-2.78	-2.16	0.42	
204	0.5	-1.87		-1.29		0.58	
205	0.53	-2.48	-3.15	-0.67	0.5	1.81	3.65
206	0.54	-2.78	-3.4	-1.73	-2.6	1.05	0.8
207	0.56	-2.99	-2.2	-1.33	0.96	1.66	3.16
208	0.56	-2.66	-1.43	-2.05	0.26	0.61	1.69
209	0.57	-3.11		-2.27		0.84	
210	0.58	0.55	-1.41	1.03	1.68	0.48	3.09
211	0.6	-2.19	-0.98	-1.5	1.1	0.69	2.08
212	0.61	-0.74	-1.93	-0.35	0.42	0.39	2.35
213	0.61	-3.86	-2.5	-2.6	-0.46	1.26	2.04
214	0.61	-2.64	0.96	-1.8	1.75	0.84	0.79
215	0.65	-3.53	-0.43	-3.03	1.87	0.5	2.3
216	0.66	-2.23	0.78	-0.89		1.34	
217	0.67	-3.18	-0.67	-2.53		0.65	
218	0.68	-1.02	-0.3	-1.06	-1.36	-0.04	-1.06
219	0.69	-2.29	-0.6	-1.63	-1.26	0.66	-0.66
220	0.7	-2.25	-1.65	-1.68	-0.1	0.57	1.55
221	0.72	-2.75	-1.14	-1.6	1.64	1.15	2.78
222	0.72	-1.18	-1.58	-0.92	-0.12	0.26	1.46
223	0.73	-2.51		-1.87	1.34	0.64	
224	0.74	-1.42	-1.78	-0.59	0.55	0.83	2.33
225	0.75	-2.46	-3.23	-1.81	0.27	0.65	3.5
226	0.75	-0.49	-1.22	-0.3	1.35	0.19	2.57
227	0.77	-2.22		-1.06	0.67	1.16	
228	0.78	-1.96	-3.22	-1.16	0.1	0.8	3.32
229	0.79	-2.47	-0.9	-2.03	-3.11	0.44	-2.21
230	0.8	-1.9	-0.03	-1.25	1	0.65	1.03
231	0.8	-2.99	-1.27	-2.86	-1.03	0.13	0.24

232	0.8	-2.69	-1.93	-2.25	-1.76	0.44	0.17
233	0.81	-1.27	-1.33	-1.23	-1.72	0.04	-0.39
234	0.82	-2.21		-1.55	0.69	0.66	
235	0.82	-2.55		-2.24		0.31	
236	0.83	-2.23	-2.32	-1.72	-0.11	0.51	2.21
237	0.83	-2.85	-1.36	-1.99	0.25	0.86	1.61
238	0.84	-5.13	-2.9	-3.28	1.64	1.85	4.54
239	0.84	-1.84	-0.62	-1.55	1.58	0.29	2.2
240	0.85	-3.48	1	-2.55	2.48	0.93	1.48
241	0.86	-3.31	-2.79	-2.65	1.06	0.66	3.85
242	0.87	-1.35	-4.13	-1.38	-1.68	-0.03	2.45
243	0.87	-3.51	-0.05	-2.63	1	0.88	1.05
244	0.88	-2.56		-2.39		0.17	
245	0.95	-0.15	-2.23	0.23	-1.43	0.38	0.8
246	0.98	-1.54	-3.39	-0.47	1.02	1.07	4.41
247	0.98	-1.92	-2.23	-1.05	-0.45	0.87	1.78
248	0.98	-2.46		-1.22	1.4	1.24	
249	1.02	-4.29	-3.99	-2.67	0.31	1.62	4.3
250	1.02	-1.69	-3.49	-1	0	0.69	3.49
251	1.03	-0.46	-1.96	0.73	2.32	1.19	4.28
252	1.06	-2.69	-3.35	-1.19	-1	1.5	2.35
253	1.09	-1.46	0.32	-0.7	0.27	0.76	-0.05
254	1.11	-2.68	-0.27	-2.42	1.49	0.26	1.76
255	1.13	-1.55	-1.35	-1.09	0.55	0.46	1.9
256	1.15	-3.58		-3.08	-1.32	0.5	
257	1.15	-1.47		0.37		1.84	
258	1.18	-3.15	-3.37	-2.23	-0.02	0.92	3.35
259	1.19	-4.27	-3.94	-2.45	-0.47	1.82	3.47
260	1.21	-2.6	-3.2	-1.22	-0.81	1.38	2.39
261	1.21	-0.54	0.37	-1.09	0.96	-0.55	0.59
262	1.24	-2.52	-3.16	-2.08	-1.81	0.44	1.35
263	1.26	-3.02	-3.44	-2.11	-1.92	0.91	1.52
264	1.3	-3.41		-3.01		0.4	
265	1.31	-2.56	-1.8	-1.98	1.86	0.58	3.66
266	1.32	-1.51		-1.26	-0.72	0.25	
267	1.34	-2.01	-1.13	-1.49	1.28	0.52	2.41
268	1.34	-1.74		-0.96		0.78	
269	1.38	-0.5	-0.49	0.3	1.6	0.8	2.09
270	1.38	-4.03	-1.36	-2.58	-0.51	1.45	0.85

271	1.4	-3.65	-4.6	-2.73	3.84	0.92	8.44
272	1.41	-1.46	-1.34	-0.05	1.81	1.41	3.15
273	1.41	-2.55	-0.66	-1.99	2.32	0.56	2.98
274	1.41	-2.49	-0.17	-1.78	1.5	0.71	1.67
275	1.41	-1.76	-2.68	-1.65	-1.84	0.11	0.84
276	1.43	-4.21	-3.94	-2.78	-1.33	1.43	2.61
277	1.43	-2.77	-1.71	-1.75	-0.45	1.02	1.26
278	1.43	-3.73	-0.2	-1.16	0.02	2.57	0.22
279	1.44	-0.62	-1.65	0.18	1.52	0.8	3.17
280	1.45	-1.76	-3.14	-1.36	1.57	0.4	4.71
281	1.45	-3.06	-2.16	-2.65	0.42	0.41	2.58
282	1.46	-2.84	-2.87	-1.92	-1.19	0.92	1.68
283	1.46	-0.78		-0.31		0.47	
284	1.49	-2.97		-2.52	-0.88	0.45	
285	1.53	-2.41		-1.79	0.49	0.62	
286	1.55	-2.27	-1.09	-1.3	1.47	0.97	2.56
287	1.57	-3.48	-4.02	-1.9	0.04	1.58	4.06
288	1.57	-2.46	0	-1.7	0.86	0.76	0.86
289	1.57	-1.8		-0.88	0.03	0.92	
290	1.58	-2.03		-1.9	-0.58	0.13	
291	1.59	-1.9	-2.99	-1.69	-0.34	0.21	2.65
292	1.6	-2.64	-5.17	-1.46	-0.1	1.18	5.07
293	1.6	-3.2	1.99	-2.97	2.48	0.23	0.49
294	1.63	-2.82	-0.33	-0.68	0.55	2.14	0.88
295	1.63	-1.22	-2.654	-0.71	-2.21	0.51	0.444
296	1.64	-5.16	-3.38	-2.52	-1.78	2.64	1.6
297	1.64	-1.53		-0.73	2.07	0.8	
298	1.66	-2.64	-5.15	-1.84	-3.7	0.8	1.45
299	1.7	-2.33	-1.26	-1.59	1.27	0.74	2.53
300	1.72	-2.95	1.23	-2.66	1.41	0.29	0.18
301	1.72	-3.07		-2.86	-0.48	0.21	
302	1.73	-2.6	1.39	-1.69	2.09	0.91	0.7
303	1.76	-4.92	-3.14	-3.85	-2.91	1.07	0.23
304	1.78	-3.49	-3.35	-2.2	0	1.29	3.35
305	1.78	0.27	-0.13	0.74	2.35	0.47	2.48
306	1.82	-1.85	-1.15	-0.81	1.21	1.04	2.36
307	1.82	-3.31	-4.37	-2.69		0.62	
308	1.83	-3.01	-1.64	-2.42	1.84	0.59	3.48
309	1.84	-1.67	1.05	-1.42		0.25	

310	1.85	1.11	-4.6	1.12	1.02	0.01	5.62
311	1.93	1.46	2.71	2.14	2.71	0.68	0
312	1.93	-2.88		-2.22		0.66	
313	1.96	-1.85	-3.6	-1.09	1.05	0.76	4.65
314	1.96	-0.73	0.65	-0.33		0.4	
315	2.03	-2.45	-3.14	-2.28	0.57	0.17	3.71
316	2.05	-2.87	-4.13	-1.78	1.17	1.09	5.3
317	2.05	-0.53		0.18	2.83	0.71	
318	2.06	-4.9		-3.84		1.06	
319	2.08	0.85	-3.06	1.17	1.71	0.32	4.77
320	2.11	-0.83	-2.85	0.3	1.91	1.13	4.76
321	2.16	-0.98	-3.35	-0.43	2.73	0.55	6.08
322	2.18	-0.28	-1.91	0.47	1.36	0.75	3.27
323	2.18	-4.22		-3.74		0.48	
324	2.19	0.42	0.16	0.72	0.77	0.3	0.61
325	2.24	-3.6		-2.94		0.66	
326	2.33	-2.99	-2.52	-2.42	-1.01	0.57	1.51
327	2.48	-1.37		-0.8	3.16	0.57	
328	2.52	-0.68	-1.65	-0.55	-1.59	0.13	0.06
329	2.55	-2.51	-1.53	-0.57	1.61	1.94	3.14
330	2.61	-0.03	-4.06	0.09	0.73	0.12	4.79
331	2.72	0.27	-2.43	0.92	1.81	0.65	4.24
332	2.74	-4.07	-2.21	-3.56	0.43	0.51	2.64
333	2.77	-0.5	-1.81	-0.07	2.2	0.43	4.01
334	2.85	-1.62	-1.05	-0.8	0.95	0.82	2
335	2.88	-1.18	-3.2	-0.45	2.97	0.73	6.17
336	2.88	-2.28	-3.43	-1.52	0.74	0.76	4.17
337	2.88	2.94	2.91	3.43	2.94	0.49	0.03
338	2.92	-2.52	-4.34	-1.24	3.01	1.28	7.35
339	2.92	-1	-2.07	-0.81	1.7	0.19	3.77
340	2.99	0.57	-2.78	2.22	1.07	1.65	3.85
341	3.07	-3.09	-3.38	-1.18	1.21	1.91	4.59
342	3.07	1.22	1.65	1.97	1.08	0.75	-0.57
343	3.18	-1.9	-4.7	0.23	0.84	2.13	5.54
344	3.3	-0.27	-1.42	0.58	2.3	0.85	3.72
345	3.37	-2.59	-1.54	-1.01	0.08	1.58	1.62
346	3.55	-3.53	1.43	-2.92	3.24	0.61	1.81
347	3.57	-0.95	-2.48	1.2	2.04	2.15	4.52
348	3.68	-0.83	0.07	-0.76	1.51	0.07	1.44

349	3.78	0.88	1.21	1.16	2.97	0.28	1.76
350	3.83	-1.55	-0.4	-0.55	2.47	1	2.87
351	4.24	-1.43	-1.75	-1	1.01	0.43	2.76
352	4.34	-2		-1.91	1.72	0.09	
353	4.36	1.57		2	2.82	0.43	
354	4.96	-3.07		-1.22		1.85	
